# Effect of exercise training on weight loss, body composition changes, and weight maintenance in adults with overweight or obesity: An overview of 12 systematic reviews and 149 studies

**DOI:** 10.1111/obr.13256

**Published:** 2021-05-06

**Authors:** Alice Bellicha, Marleen A. van Baak, Francesca Battista, Kristine Beaulieu, John E. Blundell, Luca Busetto, Eliana V. Carraça, Dror Dicker, Jorge Encantado, Andrea Ermolao, Nathalie Farpour‐Lambert, Adriyan Pramono, Euan Woodward, Jean‐Michel Oppert

**Affiliations:** ^1^ INSERM, Nutrition and obesities: systemic approaches, NutriOmics Sorbonne University Paris France; ^2^ UFR SESS‐STAPS University Paris‐Est Créteil Créteil France; ^3^ NUTRIM School of Nutrition and Translational Research in Metabolism, Department of Human Biology Maastricht University Medical Centre+ Maastricht Netherlands; ^4^ Sport and Exercise Medicine Division, Department of Medicine University of Padova Padova Italy; ^5^ Appetite Control and Energy Balance Group (ACEB), School of Psychology, Faculty of Medicine and Health University of Leeds Leeds UK; ^6^ Obesity Management Task Force (OMTF) European Association for the Study of obesity (EASO); ^7^ Department of Medicine University of Padova Padova Italy; ^8^ Faculdade de Educação Física e Desporto CIDEFES, Universidade Lusófona de Humanidades e Tecnologias Lisbon Portugal; ^9^ Department of Internal Medicine D, Hasharon Hospital, Rabin Medical Center, Sackler School of Medicine Tel Aviv University Tel Aviv Israel; ^10^ APPsyCI—Applied Psychology Research Center Capabilities and Inclusion ISPA—University Institute Lisbon Portugal; ^11^ Obesity Prevention and Care Program Contrepoids; Service of Endocrinology, Diabetology, Nutrition and Patient Education, Department of Internal Medicine University Hospitals of Geneva and University of Geneva Geneva Switzerland; ^12^ Assistance Publique‐Hôpitaux de Paris (AP‐HP), Pitié‐Salpêtrière hospital, Department of Nutrition, Institute of Cardiometabolism and Nutrition Sorbonne University Paris France

**Keywords:** body composition, exercise, weight loss, weight maintenance

## Abstract

This overview of reviews aimed to summarize the effects of exercise training programs on weight loss, changes in body composition, and weight maintenance in adults with overweight or obesity. A systematic search of systematic reviews and meta‐analyses (SR‐MAs) published between 2010 and December 2019 was performed. Only SR‐MAs of controlled trials were included. The mean difference (MD) or standardized MD (SMD) were extracted from SR‐MAs. Twelve SR‐MAs (149 studies) were included. Exercise led to a significant weight loss (4 SR‐MAs, MDs ranging from −1.5 to −3.5 kg), fat loss (4 SR‐MAs, MDs ranging from −1.3 to −2.6 kg) and visceral fat loss (3 SR‐MAs, SMDs ranging from −0.33 to −0.56). No difference in weight, fat, and visceral loss was found between aerobic and high‐intensity interval training as long as energy expenditure was equal. Resistance training reduced lean mass loss during weight loss (1 SR‐MA, MD: 0.8 [95%CI: 0.4–1.3] kg). No significant effect of exercise was found on weight maintenance (1 SR‐MA). These findings show favorable effects of exercise training on weight loss and body composition changes in adults with overweight or obesity. Visceral fat loss may lead to benefits for cardiometabolic health. More research is needed to identify training modalities that promote weight maintenance.

## 1 INTRODUCTION

Physical activity is recognized as an integral part of the management of persons with overweight or obesity in combination with diet, behavioral support, and treatment of comorbidities.^1^, ^2^, ^3^ Expected benefits of physical activity, or exercise, in this setting include positive effects on weight loss, but also fat loss together with preservation of lean mass during weight loss, as well as subsequent maintenance of weight loss.^1^, ^2^, ^3^ Well‐conducted systematic reviews and meta‐analyses published in the 2000s provided evidence of such effects of exercise on weight loss and body composition, but also acknowledged a lack of evidence on the effects of exercise on weight maintenance.^4^, ^5^, ^6^ Over the last decade, there has been growing interest in the effects of exercise on visceral adipose tissue and in the preservation of lean body mass in older adults with obesity.^7^, ^8^, ^9^, ^10^ High‐intensity interval training (HIIT) has also emerged as a promising exercise modality in adults in general as well as in those with obesity.^10^, ^11^ Finally, relations between physical activity and weight maintenance have been widely investigated although recent reviews pointed out a lack of controlled trials that would be needed to provide more robust evidence.^12^, ^13^

In response to the increasing number of systematic reviews published, overviews of reviews (also termed umbrella reviews or reviews of reviews) have been proposed as an effective strategy to provide a broad picture of evidence synthesis on a given topic.^14^ Overviews of reviews summarize existing evidence from systematic reviews without further analyses and are therefore a rapid means to inform guidelines.^15^ Given the amount of evidence published on the effects of exercise on body weight, body composition and specific fat depots such as visceral adipose tissue, an overview of reviews on these topics appears timely.

Therefore, in the context of the European Association for the Study of Obesity (EASO) Physical Activity Working Group (see summary paper for details), the aim of the present study was to conduct an overview of reviews examining the impact of exercise training programs on weight loss, changes in body composition and weight maintenance in individuals with overweight or obesity. We had a specific interest in effects on visceral adipose tissue as an outcome of exercise training and in the effects of different training modalities including HIIT.

## 2 METHODS

### 2.1 Overview of reviews

This overview of reviews follows the guidelines outlined by the Cochrane Handbook^14^ and is registered in the PROSPERO database (registration number CRD42019157823).

#### 2.1.1 Search strategy

Three electronic databases (PubMed, Web of Science and Cochrane Library) were searched for systematic reviews and meta‐analyses (SR‐MAs) published between 2010 and December 2019 using the strategy “obesity AND physical activity AND age AND weight loss” (Table S1). Limits were set to include SR‐MAs published in English. Reference lists from the resulting reviews and articles were also screened to identify additional articles.

#### 2.1.2 Study selection, inclusion, and exclusion

SR‐MAs were included in the overview if: (1) all original studies included in the SR‐MA assessed the effect of exercise training programs, that is, aerobic and/or resistance and/or high‐intensity interval training (HIIT), (2) all original studies compared either exercise training with no intervention or usual care, or exercise training in combination with other interventions (e.g., exercise + diet) with appropriate controls (e.g., diet only), (3) at least two thirds of original studies involved adults (≥18 years including older adults) with overweight (BMI ≥ 25 kg/m^2^) or obesity (BMI ≥ 30 kg/m^2^), (4) at least one of the following outcomes was reported: weight loss, fat loss, visceral fat loss, lean mass loss, or weight maintenance. Presence of obesity comorbidities such as type 2 diabetes, hypertension, dyslipidemia, metabolic syndrome, liver disease (NAFLD/NASH), and osteoarthritis was not an exclusion criterion (see summary paper for details). Abstracts and full texts were assessed for eligibility independently by two authors (AB, JMO) with uncertainty regarding eligibility discussed among authors.

#### 2.1.3 Data extraction and synthesis

Data were extracted by one author (AB) using standardized forms and then checked by a second author (JMO). The characteristics of each SR‐MA included: reference, design of original studies, population characteristics (age, gender, comorbidities), number of original studies and participants included in intervention and control groups, description of intervention (program duration, type of exercise training) and comparison, and outcomes. The findings pertaining to weight loss, fat mass loss, visceral adipose tissue loss, lean mass loss and weight maintenance of each included SR‐MA are reported as mean difference (MD) or standardized mean difference (SMD). In addition, the study author's conclusion was extracted, and an overview of the quality of the original studies and current authors' assessment of conclusion is provided. Effect sizes were considered large, medium, small or negligible when SMD was >0.8, between 0.5 and 0.8, between 0.2 and 0.5, or below 0.2, respectively.^16^ The overlap between SR‐MAs was examined for each outcome. The overlap was defined a priori as significant when more than 15% of original studies reporting the same outcome were included in two or more SR‐MAs. In this case, the SR‐MA with the highest number of original studies included, typically the most recent SR‐MA, was selected.

#### 2.1.4 Quality assessment

Study quality was assessed with a standardized tool including eight criteria, as previously described.^1^ Study quality was defined as good, fair and poor when 0, 1, or ≥2 criteria were not filled. Study quality was assessed by one author (AB) using this standardized tool and this assessment was then checked by a second author (JMO). Any disagreement between the reviewers was resolved through discussion (with a third author where necessary). The quality of original studies included in SR‐MAs was reported as assessed by the authors of SR‐MAs.

### 2.2 Additional systematic review focusing on weight maintenance

The most recent SR‐MA focusing on weight maintenance that was included in the overview was published in 2014.^17^ Therefore, an additional search for original studies published between 2010 and July 2020 was performed (see Table S1 for search terms). Inclusion criteria were as follows: (1) randomized or non‐randomized controlled trial assessing the effect of exercise training programs, (2) participants were assigned to exercise training (vs. no exercise) after an initial weight‐loss phase with the aim to compare changes in body weight during the weight maintenance phase (3), participants were adults with overweight or obesity. Study quality was assessed with a 14‐criteria form.^1^ Three of these criteria represented fatal flaws if answered “No” or “Not reported” or “Cannot determine” (i) randomization, (ii) dropout rate <20%, and (iii) intent‐to‐treat analysis. A global rating was determined based on the number of fatal flaws: good quality (0 fatal flaw), fair quality (1 fatal flaw), or poor quality (≥2 fatal flaws).

## 3 RESULTS

### 3.1 Overview of reviews

**FIGURE 1:**
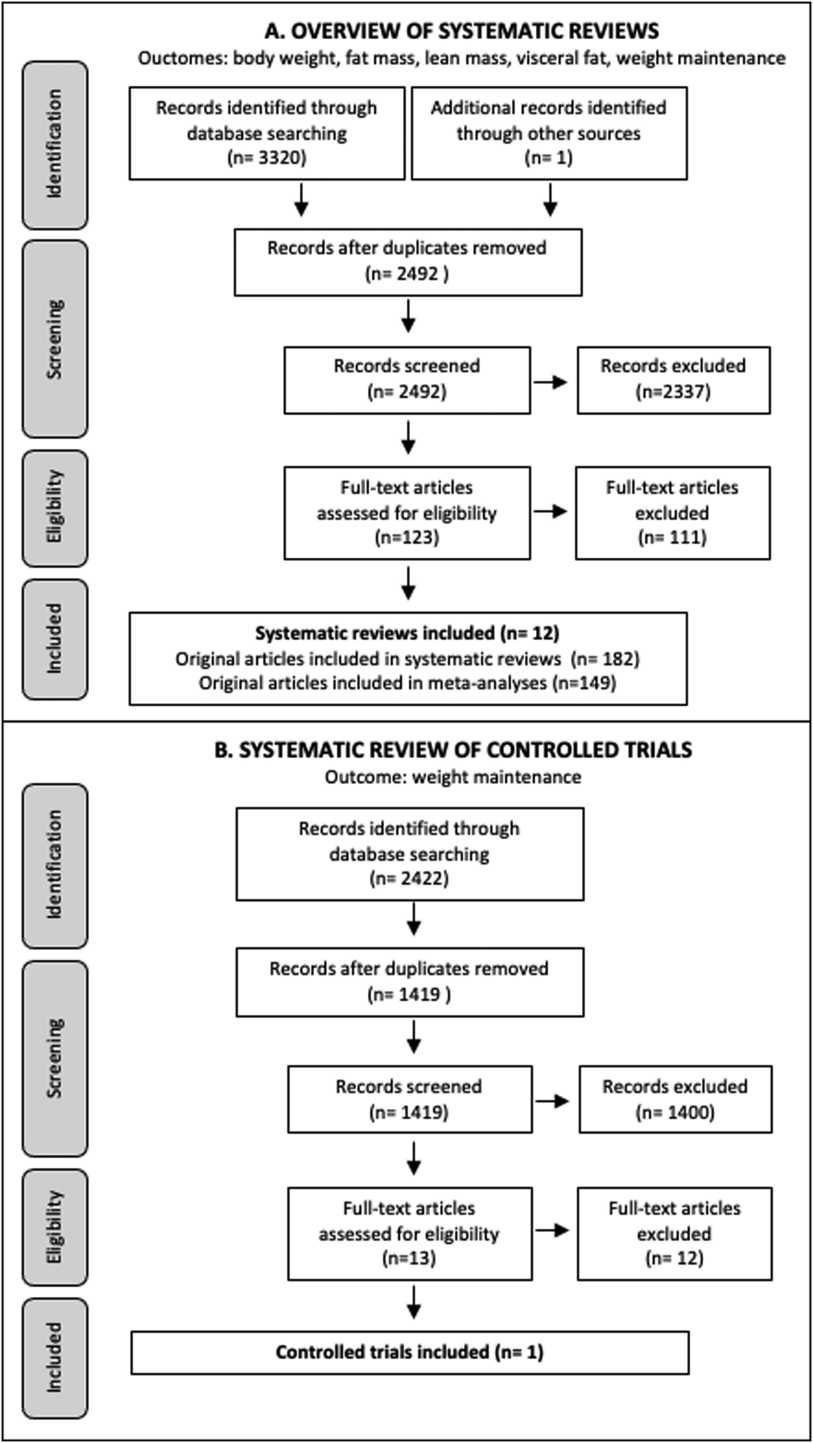
Flow diagram. (A) Overview of reviews flow diagram. (B) Systematic review flow diagram

The database search yielded 3320 articles (2492 after removing duplicates), 2337 of which were eliminated based on titles and abstracts alone (Figure 1A). The full text was retrieved from 123 reviews and 12 satisfied the inclusion criteria. From these reviews, a total of 182 unique original articles corresponded to our inclusion criteria. Among these articles, 149 were included in meta‐analyses. A total of 105, 94, 38, 37, and 3 unique original studies assessed the effect of exercise on weight loss, fat loss, lean mass/fat‐free mass loss, visceral adipose tissue loss and weight maintenance, respectively. Lean mass/fat‐free mass loss was assessed with DXA scans in 31 studies, bioelectrical impedance analysis in four studies, underwater weighing in two studies, and both DXA scans and BOD POD in one study. Of the 31 studies that used DXA, 28 and 3 studies reported the change in lean body mass and fat‐free mass, respectively. Therefore, 28 (74%) of studies reported lean body mass changes as assessed by DXA. The number of SR‐MAs included for each outcome, and the number of original articles included in these SR‐MA are presented in Table S2. The overlap between original studies ranged from 3% to 24%.

### 3.2 SR‐MAs characteristics

The characteristics of the included SR‐MAs are presented in Table 1. SR‐MAs were published between 2011 and 2019 and included randomized controlled trials (RCTs) only (N = 7) or both randomized and non‐randomized controlled trials (n = 4). One SR‐MA also included single‐group interventions but imputed data, that is, the weighted average of all available studies, when no data from a control group was available.^9^ The median (range) number of original studies included was 12 (2‐48). Most SR‐MA involved adults of both genders, one SR‐MA included post‐or peri‐menopausal females^18^ and one included older adults.^7^ Duration of exercise training ranged from 2 weeks to 12 months. Aerobic training was performed in four SR‐MAs,^19^, ^20^, ^21^, ^22^ resistance training in three SR‐MAs,^7^, ^19^, ^21^ either aerobic or resistance training or a combination of both in three SR‐MAs,^17^, ^18^, ^24^ and HIIT in four SR‐MAs.^9^, ^16^, ^23^, ^25^ Seven SR‐MAs compared exercise training with no intervention or usual care,^9^, ^16^, ^18^, ^19^, ^20^, ^22^, ^24^ five SR‐MAs compared different modalities of exercise^9^, ^19^, ^21^, ^23^, ^25^ and three SR‐MAs compared a combination of exercise and a weight‐loss diet with a weight‐loss diet only.^7^, ^17^, ^18^ Weight loss and fat mass loss were both reported in eight SR‐MAs, visceral adipose tissue loss in three SR‐MAs, lean mass loss in five SR‐MAs and weight maintenance in one SR‐MA. Except for one SR‐MA that assessed weight maintenance up to two years after initial weight loss,^17^ all SR‐MA focused on post‐intervention effect, that is, effect measured immediately after the intervention period. Findings of each included SR‐MAs are presented in Table S3.

**TABLE 1:** Characteristics of included systematic reviews

Reference	Population characteristics	Intervention and comparison	Outcomes	Number of participants
Andreato et al.^9^	*Original studies included: N = 48* ‐ Adult (18–65 years) males and females ‐N (%) studies including adults: • with overweight/obesity: 37 (81%) • inactive/untrained: 8 (17%) • with metabolic syndrome: 3 (6%)	Intervention duration: 2 to 24 weeks *Intervention*: HIIT (80–100% VO_2_max) *Control #1*: non‐exercise group *Control #2*: MICT	‐Body mass ‐Body fat (%) ‐Abdominal visceral fat area	HIIT/non‐exercise/MICT: ‐Body mass: 579/589/589 ‐Body fat: 427/406/445 ‐Visceral adipose tissue: 39/36/39
Batacan et al.^16^	*Original studies included: N = 6* ‐Adult (≥18 years) males and females ‐N (%) studies including adults: • with overweight/obesity: 4 (67%) • non‐obese adults: 2 (33%)	Intervention duration: <12 weeks *Intervention*: Short‐term HIIT (≥85% VO_2_max) *Control*: non‐exercise group	‐Body fat (%)	HIIT/control: ‐ Body fat: 68/70
Cheng et al.^18^	*Original studies included: N = 11* ‐ Peri‐ and post‐menopausal females ‐ N (%) studies including adults: • with overweight/obesity: 11 (100%)	Intervention duration: 12 weeks to 12 months *Intervention #1*: Aerobic or resistance training *Control #1*: Non‐exercise group *Intervention #2*: Weight‐loss diet + aerobic or resistance training *Control #2*: Weight‐loss diet	‐ Body mass ‐ Fat mass ‐ Lean body mass	Exercise/non‐exercise (3 studies): ‐Body mass: 215/140 ‐Fat mass: 214/140 ‐Lean body mass: 214/140Diet + exercise/diet (10 studies): ‐Body mass: 382/380 ‐Fat mass: 314/316 ‐Lean body mass: 325/346
Ismail et al.^19^	*Original studies included: N = 31* ‐Adult (≥18 years) males and females ‐N (%) studies including adults: • with overweight/obesity: 23 (74%) • non‐obese, inactive: 5 (16%) • with type 2 diabetes: 3 (10%)	Intervention duration: 8 weeks to 12 months *Intervention #1*: Aerobic training *Control #1*: Non‐exercise group *Intervention #2*: Resistance training *Control #2*: Non‐exercise group *Intervention #3*: Resistance training *Control #3*: Aerobic training	‐Visceral adipose tissue	Aerobic/control (26 studies): ‐Visceral adipose tissue: 539/533Resistance/control (13 studies): ‐Visceral adipose tissue: 355/366Aerobic/resistance (8 studies): ‐Visceral adipose tissue: 159/170
Johansson et al.^17^	*Original studies included: N = 3* ‐Adult (≥18 years) males and females ‐N (%) studies including adults: • with overweight/obesity: 3 (100%)	Intervention duration: 3 to 16 weeks *Intervention*: Initial weight‐loss period followed by aerobic (walking) or resistance training + diet counseling *Control*: Same initial weight‐loss period followed by diet counseling	‐Weight maintenance (2 years after initial weight loss)	Exercise/control: ‐Weight loss maintenance: 169/178
Mabire et al.^20^	*Original studies included: N = 22* ‐Adult (18–65 years) males and females ‐N (%) studies including adults: • with overweight/obesity: 18 (82%) • with type 2 diabetes or metabolic syndrome: 4 (18%)	Intervention duration: 10 weeks to 12 months *Intervention*: Brisk walking *Control*: Non‐exercise group	‐Body mass ‐Fat mass ‐Body fat (%) ‐Fat‐free mass	Exercise/control: ‐Body mass: 864/511 ‐Fat mass: 281/181 ‐Body fat: 707/366 ‐Fat‐free mass: 305/184
Sardeli et al.^7^	*Original studies included: N = 6* ‐Older (mean age >57 years) males and females ‐N (%) studies including adults: • with overweight/obesity: 6 (100%)	Intervention duration: 12 to 24 weeks *Intervention*: Resistance training + weight‐loss diet *Control*: Weight‐loss diet	‐Body mass ‐Fat mass ‐Lean body mass	Exercise/control: ‐Body mass: 108/184 ‐Fat mass: 108/184 ‐Lean body mass: 93/184
Schwingshackl et al.^21^	*Original studies included: N = 14* ‐Adult (≥19 years) males and females ‐N (%) studies including adults: • with overweight/obesity: 10 (71%) • inactive: 2 (14%) • with metabolic syndrome: 1 (7%) • with older adults: 1 (7%)	Intervention duration: 8 to 24 weeks *Intervention #1*: Aerobic training *Control #1*: Resistance training *Intervention #2*: Aerobic + resistance training *Control #2*: Resistance training	‐Body mass ‐Fat mass ‐Lean body mass	Aerobic/resistance (14 studies): ‐Body mass: 273/287 ‐Fat mass: 199/216 ‐Lean body mass: 173/162 Aerobic + resistance/resistance (3 studies): ‐Body mass: 82/91 ‐Fat mass: 82/91
Thorogood et al.^22^	*Original studies included: N = 5* ‐Adult (≥18 years) males and females ‐N (%) studies including adults: • with overweight/obesity: 5 (100%)	Intervention duration: 3 to 12 months *Intervention #1*: 6‐month aerobic training *Control #1*: Non‐exercise group *Intervention #2*: 12‐month aerobic training *Control #2*: Non‐exercise group	‐Body mass	6‐month aerobic/control (3 studies): ‐Body mass: 403/380 12‐month aerobic/control (2 studies): ‐Body mass: 136/129
Turk et al.^23^	*Original studies included: N = 18* ‐Adult (18–60 years) males and females ‐N (%) studies including adults: • with overweight/obesity: 15 (83%) • with metabolic syndrome or glucose intolerance: 2 (11%) • inactive: 1 (6%)	Intervention duration: 2 weeks to 6 months *Intervention #1*: HIT (≥85% maxHR) *Control #1*: MICT *Intervention #2*: HIIT (≥85% maxHR) *Control #2*: MICT	‐Body mass ‐Body fat (%)	HIT + MICT: ‐Body mass: 386 ‐Body fat (%): 296HIIT + MICT: ‐Body mass: 153 ‐Body fat (%): 157 The original studies included in the meta‐analysis were not reported. We were not able to identify the number of participants included in each group
Vissers et al.^24^	*Original studies included: N = 9* ‐Adult (≥18 years) males and females ‐N (%) studies including adults: • with overweight/obesity: 6 (67%) • with normal weight to obesity: 1 (11%) • inactive: 1 (11%) • with type 2 diabetes: 1 (11%)	Intervention duration: 10 weeks to 12 months *Intervention*: Aerobic or resistance training, or combination of both *Control*: Non‐exercise group	‐Visceral adipose tissue	Exercise/control: ‐Visceral adipose tissue: 533/457
Wewege et al.^25^	*Original studies included: N = 13* ‐Adult (18–45 years) males and females ‐N (%) studies including adults: • with overweight/obesity: 10 (77%) • inactive: 3 (23%)	Intervention duration: 5 to 16 weeks *Intervention*: HIIT (≥85% maxHR) *Control*: MICT	‐Body mass ‐Fat mass ‐Lean body mass	13 studies included N participants in HIIT/MICT: ‐Body mass: 210/205 ‐Fat mass: 180/178 ‐Lean body mass: 118/120

*Note*: Articles are presented in alphabetical order, and articles reporting results from the same trial are presented together.

Abbreviations: HIIT: high‐intensity interval training; HIT, high‐intensity training; MICT, moderate‐intensity continuous training.

### 3.3 Quality of SR‐MAs and original studies

Study quality was rated as “good,” “fair,” and “poor” in three, eight, and one SR‐MAs, respectively (Table S4). All SR‐MAs presented an adequate research question, predefined and specified eligibility criteria, used a systematic search strategy and listed the main study characteristics and results. Most SR‐MAs performed dual screening, assessed publication bias and heterogeneity, but only seven SR‐MA performed dual quality assessment. Diverse tools were used by the authors of SR‐MAs to assess the quality of original studies. Among the seven SR‐MAs that provided an overall score of study quality, the median (range) percentage of original studies with “good” or “high” quality was 23 (0 to 100)%.

### 3.4 Effectiveness of exercise training

#### 3.4.1 Weight and fat loss

All SR‐MAs reported a significant weight loss in the exercise group vs. a non‐exercise control group, whatever the type of exercise training performed, that is, aerobic training,^20^, ^22^ aerobic or resistance training or both^18^ and HIIT^9^ (Table 2). Mean weight loss ranged from −1.5 to −3.5 kg. Two out of four SR‐MAs reported a significant fat loss after aerobic training^20^ and HIIT,^9^ with a mean effect ranging from −1.3 to −2.6 kg.

**TABLE 2:**
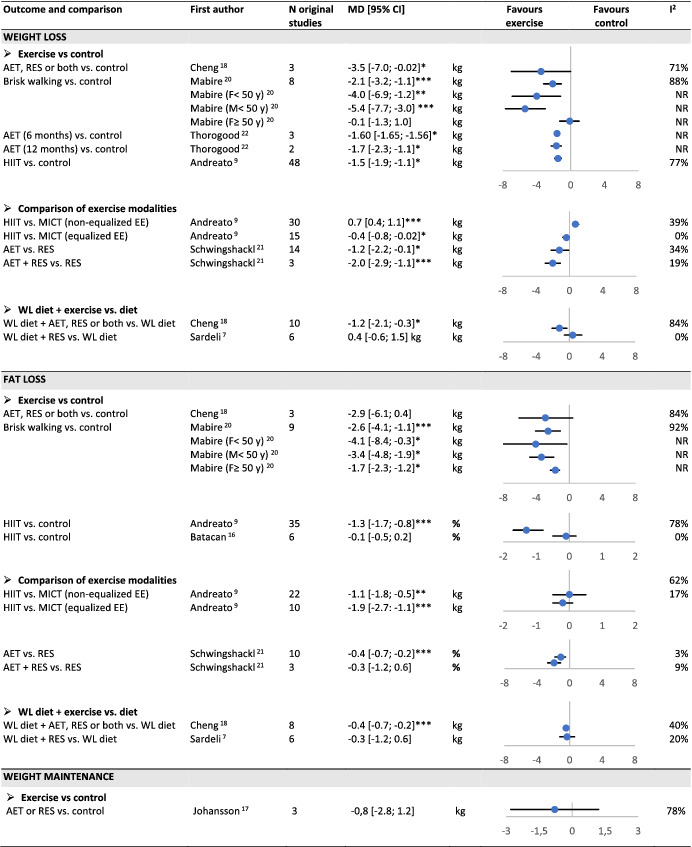
Overview of reviews on the effect of exercise training on weight loss, fat mass loss, and weight maintenance in adults with overweight or obesity

Abbreviations: AET, aerobic training; EE, energy expenditure; HIIT, high‐intensity interval training; MICT, moderate‐intensity continuous training; NR, not reported; RES, resistance training; WL diet, weight‐loss diet.

* P < 0.05;

** P < 0.01;

*** P < 0.001.

Andreato et al. directly compared HIIT and moderate‐intensity continuous training (MICT).^9^ When energy expenditure was equalized, HIIT led to a larger weight loss but, when energy expenditure was not equalized, MICT led to a larger weight loss. In both cases, the mean difference was ≤700 g. No significant difference in fat loss was observed whether energy expenditure was equalized or not. Schwingshackl et al. reported a higher weight and fat loss with aerobic training vs. resistance training, and with a combination of aerobic and resistance training vs. resistance training alone.^21^

Two SR‐MAs compared a weight‐loss diet with or without exercise. Cheng et al. included aerobic or resistance training or a combination of both and reported a significant additional weight and fat loss.^18^ Sardeli et al. focused on resistance training and found no significant effect whatever outcome.^7^

#### 3.4.2 Visceral adipose tissue loss

Three SR‐MAs reported a significant loss of visceral adipose tissue as measured by imaging techniques (CT scan or MRI) after aerobic training^19^ or after aerobic, resistance or a combination of both^24^ or HIIT (Table 3).^9^ No significant effect was found after resistance training.^19^ No significant difference was found between aerobic and resistance training (P = 0.07) and between HIIT and MICT when energy expenditure was equalized.^9^

**TABLE 3:**
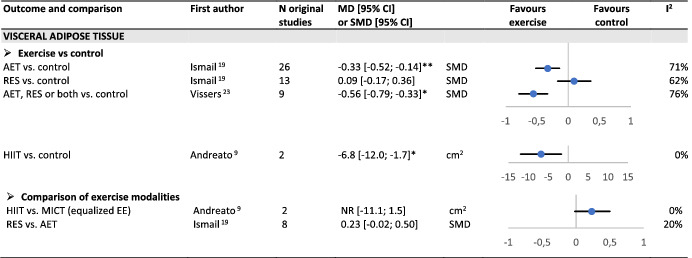
Overview of reviews on the effect of exercise training on visceral adipose tissue in adults with overweight or obesity

Abbreviations: AET, aerobic training; EE, energy expenditure; HIIT, high‐intensity interval training; MICT, moderate‐intensity continuous training; NR, not reported; RES, resistance training; WL diet, weight‐loss diet.

* P < 0.05;

** P < 0.01;

*** P < 0.001.

#### 3.4.3 Lean mass loss

Two SR‐MAs reported no significant difference in lean mass loss in an exercise group vs. a non‐exercise control group, in spite of significant weight loss in the exercisers only^18^, ^20^ (Table 4). One SR‐MA reported a lower decrease in lean body mass after resistance vs. aerobic training,^21^ and one found no difference between HIIT and MICT.^25^ During a weight loss diet, one SR‐MA reported a lower loss in lean body mass after resistance training only.^7^ Another SR‐MA also during a weight‐loss diet, found a larger loss in lean body mass with exercise training using various forms (either aerobic, resistance, or both) without analyzing the specific effect of each of them.^18^

**TABLE 4:**
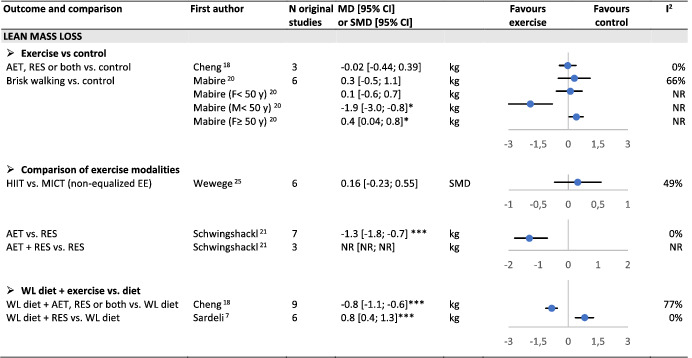
Overview of reviews on the effect of exercise training on lean mass loss in adults with overweight or obesity

Abbreviations: AET, aerobic training; EE, energy expenditure; HIIT, high‐intensity interval training; MICT, moderate‐intensity continuous training; NR, not reported; RES, resistance training; WL diet, weight‐loss diet.

* P < 0.05;

** P < 0.01;

*** P < 0.001.

#### 3.4.4 Weight maintenance

The SR‐MA included reported no significant effect of either aerobic (n = 2 studies) or resistance training (n = 2 studies) on the amount of weight regain after a low‐calorie diet^17^ (Table 2).

### 3.5 Additional systematic review focusing on weight maintenance

The search yielded 2422 articles (1419 after removing duplicates), 1400 of which were eliminated based on titles and abstracts alone (Figure 1B). The full text was retrieved from 13 articles and one satisfied the inclusion criteria.^26^ This RCT published in 2018 included 70 post‐menopausal women with overweight or obesity who, after completing a 6‐month energy‐deficit diet, were randomized into a control (n = 34) or a 12‐month weight maintenance intervention (n = 36). The intervention included two to three supervised sessions of resistance training per week. Study quality of this RCT was rated as fair. No significant difference was observed between groups for changes in body weight, fat mass, visceral adipose tissue and lean body mass during the weight maintenance phase.

## 4 DISCUSSION/CONCLUSION

This overview of reviews summarized the evidence published since 2010 on the effects of exercise on weight loss, changes in body composition and weight maintenance in adults with overweight or obesity based on 12 SR‐MAs including a total of 149 unique individual studies. Weight and fat loss were reported in the highest number of studies, that is, 105 and 94 studies, respectively. All four SR‐MAs that compared weight loss in an exercise training group vs. a non‐exercise control group reported greater weight loss of 1.5 to 3.5 kg on average in the exercise group.^9^, ^18^, ^20^, ^22^ Also, one of two SR‐MAs found an additional weight loss of about 1 kg when adding exercise to a dietary intervention, compared to the dietary intervention alone.^7^, ^27^ These results were extracted from five SR‐MAs of which four were rated as good or fair quality and three included only RCTs, and therefore provide a reasonably high degree of confidence in this overview's conclusions. They also align with literature from the previous decade^4^, ^5^, ^28^ and recent systematic reviews^29^ concluding that participation in an exercise training program does favor weight loss, although of only modest magnitude. It needs to be reminded that outcomes of meta‐analyses are average values that may mask or underestimate high inter‐individual variability in response to exercise training of weight‐related outcomes.^30^ For clinical purposes, it would be of interest to investigate further the predictors of higher weight loss with exercise.

Compared to controls without exercise, aerobic training was consistently found to be effective on weight loss, which was not the case for resistance training.^7^ Comparing one to the other, a significantly greater weight and fat loss of about 1 kg for each outcome was reported after aerobic vs. resistance training.^21^ As previously suggested, the higher energy expended through aerobic compared to resistance training might explain this difference.^4^ Interestingly, aerobic training (MICT) and HIIT led to similar weight and fat loss provided energy expenditure was equalized.^9^ Overall, these data emphasize the importance of aerobic exercise but provide no evidence on the superiority of HIIT over MICT (or the reverse) to achieve weight and fat loss, as long as the amount of energy expenditure is the same. Therefore, authors have suggested that the choice between training modalities should rely on individual preferences.^9^ While HIIT is usually considered a time‐efficient exercise strategy compared to MICT, concern has been raised about the feasibility and sustainability of HIIT in patients with obesity who seem to experience lower pleasure and enjoyment during HIIT than MICT.^31^ In addition, although HIIT is usually considered safe, even in adults with cardiovascular disease,^32^ its safety in patients with obesity has not been addressed in published SR‐MAs, which has been attributed to insufficient reporting of adverse events related to training.^9^, ^10^, ^23^ As recommended in persons with chronic diseases, a thorough medical assessment should be conducted before starting HIIT in patients with obesity, and even more so with severe obesity, to help identify any contra‐indication to participation.^33^ Importantly, no SR‐MA focused specifically on patients with severe obesity (BMI over 35 kg/m^2^) and the present findings might therefore not be adapted to such patients. In practice, the relevance of HIIT should be discussed on an individual basis after taking into account the patient's motivations and the presence of comorbidities.

This overview of reviews also provided evidence on significant effects of exercise on visceral adiposity as measured by imaging techniques such as CT scan or MRI in patients with overweight or obesity. Two SR‐MAs of high quality, and including almost exclusively RCTs, reported a significant loss in visceral adipose tissue after aerobic training or after a combination of aerobic and resistance training.^19^, ^24^ Although the effect of HIIT was assessed in only two original studies, HIIT was found to be effective at decreasing visceral adiposity compared to a control group and equally effective as MICT when energy expenditure was equalized.^9^ Resistance training was the only exercise modality that failed to significantly decrease visceral adipose tissue.^19^ When expressed in absolute values, the amount of visceral fat loss can reach 30 to 40 cm^2^.^24^ Interestingly, there is convincing evidence that reduction in visceral adipose tissue in response to exercise can be obtained in the absence of substantial weight loss.^34^, ^35^ During moderate weight loss, for example, 5% of initial body weight, the associated loss in visceral adipose tissue has been estimated at 21% in response to exercise and 13% in response to diet.^36^ Considering the specific role of visceral adiposity in increased cardiometabolic risk, especially when ≥100 cm^2^, such reduction is likely to provide important health benefits in patients with overweight or obesity.^37^

Another important aim of this overview was to summarize the effect of exercise on lean body mass in adults with overweight or obesity. Two SR‐MAs compared the change in lean mass in an exercise group and in a non‐exercise control group, and both reviews found no difference between groups despite a higher weight loss in the exercise group.^18^, ^20^ Given the well‐known reduction of lean mass after weight loss,^38^, ^39^ the lack of significant difference in lean mass change between an exercise group who experienced weight loss and a weight‐stable control group could be viewed as actual preservation of lean mass. These findings should, however, be interpreted with caution. A more common objective is to assess whether exercise training during diet‐induced weight loss helps preserve lean mass, especially in older adults with obesity.^40^ The SR‐MA by Sardeli et al.^7^ reported a significantly lower decrease in lean mass in older adults after resistance training. In contrast, when assessing various forms of exercise (aerobic, resistance or both), Cheng et al. reported a higher reduction in lean mass in the exercise group.^18^ These findings are in agreement with the extensive literature showing the superiority of resistance training over other exercise modalities to stimulate muscle protein synthesis and thus increase or maintain muscle mass.^41^

The final aim of this overview of reviews was to assess the evidence on whether exercising after weight loss may help prevent weight regain, which is so common in this setting. Contrary to our expectations, our literature search identified only one SR‐MA including three RCTs^17^ and one additional RCT published in 2018.^26^ These studies reported no evidence of a beneficial effect of exercise on weight maintenance. It should be noted that the widely cited systematic review by Washburn et al. was not included because these authors did not perform a meta‐analysis.^42^ This previous review focused on the effect of exercising during the weight loss phase (i.e., in addition to a dietary intervention) on long‐term weight loss. Seven out of nine trials included reported no significant difference in body weight between groups approximately one year after the end of the dietary intervention.^42^ Overall, these findings are in line with reviews published before 2010 stating that, at the time, well‐designed RCTs did not provide evidence on the effectiveness of physical activity for weight maintenance.^4^, ^6^ As recently summarized,^13^ this is in contrast with retrospective analyses of these RCTs that consistently showed that individuals who engaged in greater amounts of exercise experienced less weight regain, with a dose–response relationship, and that relatively large volumes of exercise (≥250 min/week) might be needed to prevent weight regain.^43^, ^44^, ^45^ A systematic analysis of weight control registries also found a consistent association between physical activity volume and weight maintenance.^46^ However, the recently published results of the METPOWeR trial that evaluated the effectiveness of three different volumes of aerobic exercise (i.e., 150, 225, and 300 min/week), in addition to a behavioral weight maintenance program, on the prevention of weight regain over 12 months after a ≥5% weight loss showed no significant difference in weight regain across exercise groups.^47^ Overall, 88% of participants across groups maintained a 5% weight loss at 12 months, and the mean weight regain ranged from 1.1 kg in the low‐volume exercise group to 2.8 kg in the high‐volume exercise group.^47^ Authors of this study conclude that minimal volume of exercise may favor successful weight maintenance, although the study was not designed to show equivalence between interventions.^47^ Poor adherence to exercise protocols has been cited as the main reason why RCTs fail to demonstrate an effect of exercise on weight maintenance.^6^, ^13^, ^47^ Finding effective strategies to improve patients' adherence to exercise is therefore required to provide more robust evidence on the effect of exercise on weight maintenance in participants with overweight or obesity, a crucial issue in long‐term obesity management.

### 4.1 Limitations

This overview of reviews synthetizes the literature on the effect of exercise training programs on weight loss, body composition changes including visceral adipose tissue, and weight maintenance. Two important research questions were not addressed in this overview. First, we did not assess the effect of exercise according to the duration of the program. Even though the program duration was highly variable across original studies included in the various SR‐MAs (e.g., from 2 weeks to 6 months in the SR‐MA by Andreato et al.^9^), only one SR‐MA stratified the analysis by duration of the program.^22^ A mean weight loss of 1.6 and 1.7 kg was reported after a 6‐month or a 12‐month aerobic training program, respectively, suggesting no effect of the program duration. Second, we did not assess the effect of weekly volume of exercise. None of the SR‐MAs included in this overview addressed this question. The duration of exercise ranged from 60^48^ to 440^49^ min per week, with most studies assessing programs based on 150 to 200 min per week of exercise.

### 4.2 Conclusion

This overview of reviews provides evidence that exercise training improves body weight and body composition in adults with overweight or obesity. The benefits of exercise include reductions of body weight, total body fat and visceral adipose tissue. Although the effect on weight and fat loss is of relatively small magnitude (only a few kilograms difference), the reduction of visceral fat is likely to enhance cardiometabolic health in these patients. Importantly, visceral fat loss can occur even when participants experience small or no weight loss. Regarding the outcomes of weight, fat, and visceral fat loss, aerobic training is more effective than resistance training, with HIIT being as effective as MICT at the same level of energy expenditure. During diet‐induced weight loss, resistance training is the most effective exercise modality to preserve lean body mass. In contrast with widely held views, in line with reviews from the previous decade (before 2010), this overview does not provide evidence that exercise helps prevent weight regain after weight loss. Overall, considering the specific effects of aerobic and resistance training on one side, the well‐proven benefits of HIIT on the other, but also the lack of data on the safety of HIIT in adults with obesity, we recommend the careful assessment of initial health status to precisely define clinical objectives and to take into account individual preferences when designing exercise training programs in adults with overweight or obesity.

## CONFLICT OF INTEREST

No conflict of interest statement.

## AUTHOR CONTRIBUTIONS

AB and JMO performed the literature search, study selection, data extraction, and quality assessment. All authors participated in the interpretation of data. AB and JMO drafted the manuscript, and all authors critically revised the manuscript.

## Supporting information

**Table S1.** Keywords included in database search strategy
**Table S2**. Number of original studies included for each overview
**Table S3**. Findings of systematic reviews included in the overview
**Table S4**. Summary of quality assessment of systematic reviews
