# Supplementary material for: Effect of exercise training on weight loss, body composition changes, and weight maintenance in adults with overweight or obesity: An overview of 12 systematic reviews and 149 studies
Source: Obes Rev. 2021 May 6;22(Suppl 4):e13256. doi: 10.1111/obr.13256 (PMC8365736; doi:10.1111/obr.13256)
Supplement: Supplementary file 1 — Table S1. Keywords included in database search strategy Table S2. Number of original studies included for each overview Table S3. Findings of systematic reviews included in the overview Table S4. Summary of quality assessment of systematic reviews [file OBR-22-e13256-s001.pdf]

SUPPORTING INFORMATION

Effect of exercise training on weight loss, body composition changes and weight maintenance in adults with overweight or obesity: An overview of 12 systematic reviews and 149 studies

Alice Bellicha*[1,2], Marleen A. van Baak[3], Francesca Battista[4], Kristine Beaulieu[5], John E. Blundell[5], Luca Busetto[6,7], Eliana V. Carraça[8], Dror Dicker[6,9], Jorge Encantado[10], Andrea Ermolao[4], Nathalie Farpour-Lambert[6,11], Adriyan Pramono[3], Euan Woodward[6], Jean-Michel Oppert[12]

*Corresponding Author

Alice Bellicha, PhD

Service de Nutrition, Hôpital Pitié-Salpêtrière

47-83 Boulevard de l'Hôpital

75013 PARIS, France

Tel: +33(1)42175782

Fax:+33(1)42175963

E-mail: alice.bellicha@u-pec.fr

Table S1. Keywords included in database search strategy

Obesity	Physical activity	Age	Weight loss	Weight maintenance
Overweight<br>Obesity<br>Obese	Physical activit*<br>Exercise<br>Sport<br>Endurance activit*<br>Aerobic activit*<br>Cardiovascular activit*<br>Resistance training<br>Strength training<br>Muscle-strengthening<br>Weight-Lifting program<br>High-intensity interval training<br>HIIT<br>Physical conditioning<br>Walking<br>Sedentary time<br>Sedentary lifestyle<br>Sitting time	Adults<br>(NOT child, children, adolescents, pediatric)	Weight loss<br>Fat loss<br>Lean loss<br>Lean body loss<br>Weight maintenance<br>Weight regain	Weight maintenance<br>Weight regain<br>Weight loss maintenance<br>Maintenance of weight regain

Table S2. Number of original studies included for each overview

Overview topic	Number of included SR-MA	Years of publication	Total number of original studies	Number of unique original studies	Original study overlap
**Weight loss**					
Exercise vs. control	4	2011-2019	68	66	2.9%
Weight-loss diet + exercise vs. weight-loss diet	2	2018	16	14	12.5%
HIIT vs. aerobic training	3	2017-2019	75	60	20%
Aerobic vs. resistance	1	2013	--	14	--
Aerobic + resistance vs. resistance	1	2013	--	3	--
**Fat mass loss**					
Exercise vs. control	4	2017-2018	53	49	7.5%
Weight-loss diet + exercise vs. weight-loss diet	2	2018	14	12	14.3%
HIIT vs. aerobic training	3	2017-2019	64	51	24.1%
Aerobic vs. resistance	1	2013	--	8	--
Aerobic + resistance vs. resistance	1	2013	--	3	
**Visceral adipose tissue loss**					
Exercise vs. control	3	2012-2019	42	37	11.9%
Aerobic vs. resistance	1	2012	--	14	--
**Lean body mass loss**					
Exercise vs. control	2	2017-2018	9	8	11.1%
Diet + exercise vs. diet	2	2018	15	13	13.3%
HIIT vs. aerobic training	1	2017	--	6	--
Aerobic vs. resistance	1	2013	--	7	--
**Weight loss maintenance**					
Exercise vs. control	1	2014	--	3	--

HIIT, high-intensity interval training

Table S3. Findings of systematic reviews included in the overview

Quality of original studies	Findings			Review author's conclusion	Overview authors' assessment of conclusions
**Andreato 2019** [1]					
**Study quality**:<br>-Score 5/5: 8/48 (17%)<br>-Score 4/5: 2/48 (4%)<br>-Score 3/5: 19/48 (40%)<br>-Score 2/5: 15/48 (31%)<br>-Score 1/5: 4/48 (8%)<br>-Mean (SD) score: 2.9 (1.2)<br>Tool used for rating quality:<br>TESTEX scale<br>**Design of included studies**:<br>RCT: 37/48 (77%)<br>Non-RCT: 5/48 (10%)<br>Single-group intervention: 4/48 (8%)<br>Not written in English: 2/48 (4%)	**HIIT vs control group**<br>Body mass (kg)<br>Body fat (%)<br>Visceral adipose tissue (cm^2)	**MD [95%CI] (P-value)**<br>-1.45 [-1.85; -1.05] (NR)<br>-1.29 [-1.70; -0.87] (NR)<br>-6.8 [-12.0; -1.7] (NR)	**I^2 (P-value)**<br>77% (P< 0.001)<br>78% (P< 0.001)<br>0% (P= 0.43)	*"HIIT is effective in reducing body mass (…) body fat percentage and abdominal visceral fat area"*<br>*" (…) The magnitude that can be considered is modest"*<br>*"Although some differences between HIIT and MICT were found, when equalization of the sessions between the two training methods was considered, the only difference remaining was for body mass"*<br>*"HIIT can be considered an effective training method for the treatment of obesity, but its superiority in relation to MICT should be viewed with reservation"*<br>*"Study quality is a limiting factor of this meta-analysis". "Another important limiting aspect was the lack of control of the participants' diets"*	Appropriate conclusions based on available data.<br>As reported by the authors, only 21% studies scored 4 or 5 on the TESTEX scale.<br>Most studies had small sample sizes (about 8 to 15 in each group).<br>Some studies did not include a control group and data were imputed to include these studies in the meta-analyses.<br>The effect on body mass was modest (-1.45 kg vs control group), which could have been more strongly emphasized by the authors.<br>The major strength of this study was to compare HIIT and MICT with equal energy expenditure.
	**HIIT vs MICT (all studies)**<br>Body mass (kg)<br>Body fat (%)<br>Visceral adipose tissue (cm^2)	**MD [95%CI] (P-value)**<br>0.40 [0.09; 0.72] (NR)<br>-0.12 [-0.49; 0.19] (NR)<br>NR [-11.1; 1.5] (NR)	**I^2 (P-value)**<br>40% (P= 0.002)<br>55% (P< 0.001)<br>0% (P= 0.65)		
	**HIIT vs MICT**<br>**(EE not equalized)**<br>Body mass (kg)<br>Body fat (%)<br>Visceral adipose tissue (cm^2)	**MD [95%CI] (P-value)**<br>0.72 [0.35; 1.10] (P= 0.0002)<br>-0.0 [-0.49; 0.49] (P= 1)<br>NR [NR] (NR)	**I^2 (P-value)**<br>39% (P= 0.009)<br>62% (P< 0.001)<br>NR (NR)		
	**HIIT vs MICT**<br>**(EE equalized)**<br>Body mass (kg)<br>Body fat (%)<br>Visceral adipose tissue (cm^2)	**MD [95%CI] (P-value)**<br>-0.41 [-0.79; -0.02] (P= 0.01)<br>-0.22 [-0.52; 0.08] (P= 0.44)<br>NR [-11.1; 1.5] (NR)	**I^2 (P-value)**<br>0% (P= 0.97)<br>17% (P= 0.29)<br>0% (P= 0.65)		
	**Meta-regression**				
	For changes in body mass	Significant regression for:<br>- number of sessions (favors more sessions: P= 0.004)<br>- exercise mode (favors running: P= 0.035)<br>- age (favors younger: P= 0.03)<br>- sex (favors men: P< 0.0001)			
	For changes in WC	Significant regression for:<br>- exercise mode (favors running: P= 0.023)			
	For changes in body fat	Significant regression for:<br>- age (favors older: P= 0.009)			
	For changes in visceral adipose tissue	Significant regression for:<br>- study quality (favors good-quality studies: P= 0.04)			
**Batacan 2017** [2]					

**Study quality**:<br>-High: 0 (0%)<br>-Fair: 5/6 (83%)<br>-Low: 1/6 (17%)<br>Tool used for rating quality:<br>Downs & Blake scale (modified)<br>**Design of included studies**:<br>RCT: 5/6 (83%)<br>Non-RCT: 1/6 (17%)	**HIIT vs control**<br>Body fat	**SMD [95%CI] (P-value)**<br>-0.14 [-0.48; 0.20] (P= 0.42)	**I^2 (P-value)**<br>0% (P= 1)	*"These findings suggest that HIIT is an effective stimulus for reducing body fat levels (even in the absence of weight loss) for those individuals with large fat mass"*<br>*"Most studies included used relatively small sample sizes"*	This statement applies only to long-term HIIT interventions (> 12 weeks).<br>These results were not included in this overview of reviews because less than 67% of studies fit with inclusion criteria.<br>The control group was either a non-exercise group or MICT, which limits the validity of findings.<br>The total number of participants (about 70 in each group) was very limited.
**Cheng 2018** [3]					
**Study quality**:<br>Low risk of bias:<br>-Randomized allocation sequence: 8/11 (73%)<br>-Allocation concealment: 2/11 (18%)<br>-Blinding of participants and personal: 1/11 (9%)<br>-Blinding and completeness of outcome data as adequate: 1/11 (9%)<br>-Incomplete outcome data: 10/11 (91%)<br>-Selective reporting: 5/11 (46%)<br>Tool used for rating quality:<br>Cochrane risk of bias assessment tool<br>**Design of included studies**:<br>RCT: 8/11 (73%)<br>Non-RCT: 3/11 (27%)	**Exercise vs control group**<br>Body mass (kg)<br>Fat mass (kg)<br>Lean body mass (kg)<br>**Diet + exercise vs diet**<br>Body mass (kg)<br>Fat mass (kg)<br>Lean body mass (kg)	**MD [95%CI] (P-value)**<br>-3.49 [-6.96; -0.02] (P= 0.049)<br>-2.85 [-6.09; 0.40] (P= 0.09)<br>-0.02 [-0.44; 0.39] (P= 0.92)<br>**MD [95%CI] (P-value)**<br>-1.22 [-2.14; -0.30] (P= 0.01)<br>-0.44 [-0.67; -0.21] (P< 0.001)<br>-0.84 [-1.13; -0.55] (P< 0.001)	**I^2 (P-value)**<br>70.5% (P= 0.034)<br>84.1% (P= 0.002)<br>0% (P= 0.830)<br>**I^2 (P-value)**<br>83.8% (P< 0.001)<br>39.7% (P= 0.11)<br>76.5% (P< 0.001)	*"Exercise interventions alone resulted in greater reductions in body weight loss than seen in control groups, but no difference between the two groups was found in change of fat mass loss and lean mass loss after the intervention. Studies applying dietary plus exercise interventions demonstrated greater efficacy than dietary interventions alone"*<br>*"Results of this study are limited by the relatively small number of studies included in the analysis and the low number of participants per study and overall"*	Appropriate conclusions based on available data.<br>Diverse dietary and exercise interventions were assessed, which might explain the statistical heterogeneity.<br>This study was conducted in peri- and post-menopausal women, and results cannot be generalized to other groups of subjects with obesity
**Ismail 2012** [4]					
**Study quality**:<br>-High: 28/29 (97%)<br>-Fair: 1/29 (3%)<br>-Low: 0/29 (0%)<br>Tool used for rating quality:<br>Downs & Blake scale (modified)<br>**Design of included studies**:<br>RCT: 29/29 (100%)	**Aerobic vs control group**<br>Visceral adipose tissue<br>**Resistance vs control group**<br>Visceral adipose tissue<br>**Resistance vs aerobic**<br>Visceral adipose tissue	**SMD [95%CI] (P-value)**<br>-0.33 [-0.52; -0.14] (P< 0.01)<br>**SMD [95%CI] (P-value)**<br>0.09 [-0.17; 0.36] (P= 0.49)<br>**SMD [95%CI] (P-value)**<br>0.23 [-0.02; 0.50] (P= 0.07)	**I^2 (P-value)**<br>71.0% (P< 0.001)<br>**I^2 (P-value)**<br>61.7% (P< 0.01)<br>**I^2 (P-value)**<br>20.1% (P= 0.26)	*"When compared with a control intervention, aerobic exercise (AEx) therapy is effective in lowering VAT. Progressive resistance training (PRT) itself failed to induce significant reduction in VAT when compared with the control group. In studies where AEx and PRT were directly compared, the effect size favoured*	Appropriate conclusions based on available data.<br>Strengths of the study: only RCT, only studies assessing VAT with CT or MRI were included<br>The tool used for assessing study quality was modified and adapted to physical activity interventions, which might have overestimated study quality

				AEx training but did not reach statistical significance”	
**Johansson 2014** [5]					
**Study quality**:<br>-Extent of loss to follow-up: NR<br>-Adequacy of randomization and allocation concealment: NR<br>-Blinding of participants, data collectors and outcome assessors: NR<br>Tool used for rating quality: NR<br>**Design of included studies**:<br>RCT: 3/3 (100%)	**Exercise vs control**<br>Weight loss maintenance (kg)	**MD [95%CI] (P-value)**<br>-0.8 [-2.8; 1.2] kg (NR)	**I^2 (P-value)**<br>78% (P< 0.001)	*“Exercise was not associated with improved (weight loss) maintenance”*	When analyzing only the 2 studies that assess exercise training only, the effect on weight loss maintenance was significant, although modest Only RCT were included but the number of studies and participants included was very limited.
**Mabire 2017** [6]					
**Study quality**:<br>-High: 4/22 (18%)<br>-Moderate: 13/22 (59%)<br>-Poor: 5/22 (23%)<br>Tool used for rating quality:<br>Delphi score<br>**Design of included studies**:<br>RCT: 22/22 (100%)	**Exercise vs control (Pooled analysis)**<br>Body mass (kg)<br>Fat mass (kg)<br>Body fat (%)<br>Fat-free mass (kg)<br>**Exercise vs control (Male < 50 y)**<br>Body mass (kg)<br>Fat mass (kg)<br>Body fat (%)<br>Fat-free mass (kg)<br>**Exercise vs control (Female < 50 y)**<br>Body mass (kg)<br>Fat mass (kg)<br>Body fat (%)<br>Fat-free mass (kg)<br>**Exercise vs control (Female > 50 y)**<br>Body mass (kg)<br>Fat mass (kg)<br>Body fat (%)<br>Fat-free mass (kg)	**MD [95%CI] (P-value)**<br>-2.1 [-3.2; -1.1] (P< 0.0001)<br>-2.6 [-4.1; -1.1] (P= 0.0009)<br>-1.4 [-2.2; -0.6] (NR)<br>0.3 [-0.5; 1.1] (NR)<br>**MD [95%CI] (P-value)**<br>-5.4 [-7.7; -3.0] (P< 0.00001)<br>-3.4 [-4.8; -1.9] (NR)<br>-3.0 [-4.4; -1.7] (NR)<br>-1.9 [-3.0; -0.8] (NR)<br>**MD [95%CI] (P-value)**<br>-4.0 [-6.9; -1.2] (P= 0.005)<br>-4.1 [-8.4; -0.3] (NR)<br>-2.7 [-3.5; -1.8] (NR)<br>0.1 [-0.6; 0.7] (NR)<br>**MD [95%CI] (P-value)**<br>-0.1 [-1.3; 1.0] (P= 0.84)<br>-1.7 [-2.3; -1.2] (NR)<br>-0.3 [-1.3; 0.8] (NR)<br>0.4 [0.04; 0.8] (NR)	**I^2 (P-value)**<br>88% (P< 0.0001)<br>92% (P< 0.0001)<br>96% (NR)<br>66% (NR)<br>**I^2 (P-value)**<br>NR (NR)<br>NR (NR)<br>NR (NR)<br>NR (NR)<br>**I^2 (P-value)**<br>NR (NR)<br>NR (NR)<br>NR (NR)<br>NR (NR)<br>**I^2 (P-value)**<br>NR (NR)<br>NR (NR)<br>NR (NR)<br>NR (NR)	*“There is low quality evidence to support that a 12-16 week, moderate intensity brisk walking intervention can create a clinically significant reduction in fat mass in obese adults whilst preserving fat-free mass without a dietary intervention”*<br>*“The meta-analysis for change in body weight suggests that men and women under fifty years old attain a clinically significant 5% weight loss, but the overall result was mediated by women over fifty years, who comprised the majority of the study populations and who neither lost or gained weight”*	Appropriate conclusions based on available data.
**Sardeli 2018** [7]					
**Study quality**:<br>-Good: 3/6 (50%)<br>-Fair: 3/6 (50%)	**Exercise + caloric restriction vs caloric restriction**<br>Body mass (kg)	**MD [95%CI] (P-value)**<br>0.4 [-0.6; 1.5] (P= 0.44)	**I^2 (P-value)**<br>0% (P= 0.56)	*“Resistance training + caloric restriction prevents 93% of the lean body mass loss induced by caloric*	Appropriate conclusions based on available data.

<u>Tool used for rating quality</u>:<br>PEDro scale<br>**Design of included studies**:<br>RCT: 6/6 (100%)	Fat mass (kg)<br>Lean body mass (kg)	-0.3 [-1.2; 0.6] (P= 0.71)<br>0.8 [0.4; 1.3] (P< 0.001)	20% (P= 0.28)<br>0% (P= 0.94)	*restriction although it does not affect body mass and fat body mass reductions as compared to caloric restriction without resistance training."*	Only RCTs were included but the number of studies and participants was limited.
**Schwingshackl 2013** [8]					
**Study quality**:<br>Low risk of bias:<br>-Random sequence generation: 4/14 (29%)<br>-Allocation concealment: 1/14 (7%)<br>-Blinding of participants and personnel: 0/14 (0%)<br>-Incomplete outcome data: > 75% (details not reported)<br>-Selective reporting: 14/14 (100%)<br>-Systematic difference in care: > 75% (details not reported)<br><u>Tool used for rating quality</u>:<br>Cochrane risk of bias assessment tool<br>**Design of included studies**:<br>RCT: 14/14 (100%)	**Aerobic vs resistance training**<br>Body mass (kg)<br>Fat mass (kg)<br>Lean body mass (kg)<br>**Aerobic + resistance vs resistance training**<br>Body mass (kg)<br>Fat mass (kg)<br>Lean body mass (kg)	**MD [95%CI] (P-value)**<br>-1.2 [-2.2; 0.1] (P= 0.04)<br>-1.1 [-1.8; -0.5] (P= 0.001)<br>-1.3 [-1.8; -0.7] (P< 0.00001)<br>**MD [95%CI] (P-value)**<br>-2.0 [-2.9; -1.1] (P< 0.0001)<br>-1.9 [-2.7: -1.1] (P< 0.00001)<br>NR [NR] (NS)	**I^2 (P-value)**<br>34% (P= 0.03)<br>3% (P= 0.28)<br>0% (P= 0.223)<br>**I^2 (P-value)**<br>19% (P= 0.29)<br>9% (P= 0.85)<br>NR (NR)	*"Aerobic exercise training (AET) is more efficient in reducing body weight and fat mass when compared to resistance training (RT). However, RT turned out to be more suitable when it comes to an improvement of lean body mass"*<br>*"Combined training was more powerful in reducing body weight or fat mass when compared to RT"*	Appropriate conclusions based on available data.<br>Only RCT were included but the number of participants included in limited.
**Thorogood 2011** [9]					
**Study quality***:<br>Low risk of bias:<br>-Random sequence generation: 1/14 (7%)<br>-Allocation concealment: 3/14 (21%)<br>-Blinding of outcomes: 4/14 (29%)<br>-Incomplete outcome data: 7/14 (50%)<br>-Selective reporting: 13/14 (93%)<br><u>Tool used for rating quality</u>:<br>Cochrane risk of bias assessment tool<br>**Design of included studies**:<br>RCT: 6/6 (100%)	**6-month aerobic training vs control**<br>Body mass (kg)<br>**12-month aerobic training vs control**<br>Body mass (kg)	**MD [95%CI] (P-value)**<br>-1.60 [-1.65; -1.56] (NR)<br>**MD [95%CI] (P-value)**<br>-1.7 [-2.3; -1.1] (NR)	**I^2 (P-value)**<br>NR (NR)<br>**I^2 (P-value)**<br>NR (NR)	*"We found that aerobic exercise programs of moderate intensity, with durations ranging from 12 weeks to 12 months, resulted in modest weight reduction"*	Appropriate conclusions based on available data, although 12-week interventions were not included in the meta-analysis.<br>Only RCTs and ITT trials were included, which strengthens findings of this meta-analysis. However, very few studies were included and meta-analyses were performed with only 2 or 3 studies for each outcome.
**Turk 2017** [10]					
**Study quality**:<br>Low risk of bias:<br>-Random sequence generation: 3/18 (17%)<br>-Allocation concealment: 2/18 (11%)	**HIT vs MICT**<br>Body mass (kg)<br>Body fat (%)<br>**HIIT vs MICT**<br>Body mass (kg)	**MD [95%CI] (P-value)**<br>-1.2 [-4.2; 1.8] (P= 0.44)<br>-1.7 [-3.1; -0.3] (P= 0.02)<br>**MD [95%CI] (P-value)**<br>-0.4 [-5.3; 4.5] (P= 0.87)	**I^2 (P-value)**<br>0% (NR)<br>30% (P= 0.17)<br>**I^2 (P-value)**<br>7% (NR)	*"This meta-analysis showed a significant reduction in the percentage of body fat in favour of HIT compared to traditional exercise. Moreover, HIIT showed the same*	Appropriate conclusions based on available data.<br>The lack of matching on energy expenditure between HIT/HIIT and

-Blinding of participants and personnel: 0/18 (0%)<br>-Blinding of outcomes: 3/18 (17%)<br>-Incomplete outcome data:8/18 (44%)<br>-Selective reporting: 2/18 (11%)<br><u>Tool used for rating quality</u>:<br>Cochrane risk of bias assessment tool<br>**Design of included studies**:<br>RCT: 18/18 (100%)	Body fat (%)<br>**Meta-regression**<br>For changes in body fat	-2.0 [-3.7; -0.3] (P= 0.02)<br><br>No significant regression was found for:<br>- duration of intervention<br>- intensity of intervention<br>- intervals<br>- number of repetitions<br>- baseline BMI<br>- younger age<br>- gender	0% (NR)	*effect compared to lower intensity continuous exercise."*<br>*"However, there was no difference in the amount of weight loss between HIT or traditional exercise"*<br>*"Most included studies did not provide data on equal energy expenditure between HIT and traditional exercise forms"*<br>*"HIT is feasible and well tolerated in persons with obesity"*	MICT appears to be a main limitation to compare these forms of exercise<br>Secondary effects were not directly assessed in this meta-analysis.<br>Only RCTs were included, which strengthens findings of this meta-analysis.
**Vissers 2013** [11]					
**Study quality**:<br>Score >10/15: 9/9 (100%)<br><u>Tool used for rating quality</u>:<br>The Critical Review Form for Quantitative Studies<br>**Design of included studies**:<br>RCT: 8/9 (89%)<br>NRCT: 1/9 (11%)	**Exercise vs control**<br>Visceral adipose tissue<br><br>Females<br>Males	**SMD [95%CI] (P-value)**<br>-0.56 [-0.79; -0.33] (NR)<br>**MD [95%CI] (P-value)**<br>-37.1 cm^2 [NR]<br>-46.5 cm^2 [NR]	**I^2 (P-value)**<br>76% (P< 0.001)	*"This meta-analysis showed that a decrease of visceral adipose tissue can be obtained by exercise without diet in people with overweight and obesity."*<br>*"Based on the Hedge's g, it seems that the 5 controlled clinical trials that used cm^2 as unit for VAT, slightly overestimate the effect of exercise on reduction of VAT compared to the total of 9 controlled clinical trials (-0.630 versus -0.561). Taking that into account, the results of this meta-analysis show that exercise without diet has the potential to reduce VAT with >30 cm^2 in females and >40 cm^2 in males"*	Appropriate conclusions based on available data.<br>Included only exercise-only interventions
**Wewege 2017** [12]					
**Study quality**:<br>-Good: 3/13 (23.1%)<br>-Fair: 7/13 (53.8%)<br>-Poor: 3/13 (23.1%)<br><u>Tool used for rating quality</u>*****:<br>PEDro scale<br>**Design of included studies**:<br>RCT: 11/13 (85%)<br>NRCT: 2/13 (15%)	**HIIT vs MICT**<br>Body mass<br>Fat mass<br>Lean body mass	**SMD [95%CI] (P-value)**<br>0.09 [-0.10; 0.28] (P= 0.38)<br>0.03 [-0.18; 0.24] (P= 0.79)<br>0.16 [-0.23; 0.55] (P= 0.42)	**I^2 (P-value)**<br>0% (P= 0.51)<br>0% (P= 0.97)<br>49% (P= 0.08)	*"Considering HIIT shows similar efficacy to MICT, but with ~40% less time commitment each week, HIIT can be considered a time-efficient alternative for managing overweight and obese individuals."*<br>*"About 10 weeks of high-intensity or moderate-intensity exercise training can reduce body fat by about 2 kg in the absence of body mass changes."*	Appropriate conclusions based on available data.<br>Authors of the review presented within-groups changes in body mass and composition in both HIIT and MICT interventions. We did not report these findings because no comparison was made with a control group.

"The quality of included studies and the small pooled sample size (total of 424 adults) present limitations for this analysis"	Safety of HIIT was not assessed in subjects with overweight or obesity this review.

- In the review by Andreato et al., when no data from a control group were available, the weighted average of all available studies was considered for group imputation
- In the review by Ismail et al., the authors did not calculate an overall score of study quality. We applied the rating system used in the review by Batacan et al. to rate the overall study quality of the original studies: the score obtained was divided by the maximum possible score (i.e. 18) and multiplied by 100 to provide a study quality percentage. Study quality percentages were classified as high (≥ 66.7%), fair (50-66.6%), and low (< 50%).
- In the review by Sardeli et al., the authors report the PEDro score. We applied the standard rating system of the PEDro scale (https://www.pedro.org.au/) to rate the study quality: score < 4 classified as poor-quality, 4–5 as fair-quality, 6–8 as good-quality and ≥ 9 as excellent-quality.
- In the study by Thorogood et al. (2011): the study quality was reported for all 14 studies included in the systematic review, and not not reported specificallu for the 6 studies included in the meta-analysis
- In the review by Wewege et al., the authors report the PEDro score. We applied the standard rating system of the PEDro scale (https://www.pedro.org.au/) to rate the study quality: score < 4 classified as poor-quality, 4–5 as fair-quality, 6–8 as good-quality and ≥ 9 as excellent-quality.

NR, not reported

Table S4. Summary of quality assessment of systematic reviews

**References**	**Criteria**								**Total "Yes"**	**Total "No"**	**Total "other"**	**Quality rating**
	**1**	**2**	**3**	**4**	**5**	**6**	**7**	**8**				
Andreato 2019 [1]	Yes	Yes	Yes	Yes	Yes	Yes	No	Yes	7	1	0	Fair
Batacan 2017 [2]	Yes	Yes	Yes	Yes	Yes	Yes	No	Yes	7	1	0	Fair
Cheng 2018 [3]	Yes	Yes	Yes	Yes	NR	Yes	Yes	Yes	7	0	1	Fair
Ismail 2012 [4]	Yes	Yes	Yes	No	Yes	Yes	Yes	Yes	7	1	0	Fair
Johansson 2014 [5]	Yes	Yes	Yes	Yes	No	Yes	Yes	Yes	7	1	0	Fair
Mabire 2017 [6]	Yes	Yes	Yes	No	Yes	Yes	Yes	Yes	7	1	0	Fair
Sardeli 2018 [7]	Yes	Yes	Yes	Yes	No	Yes	Yes	Yes	7	1	0	Fair
Schwingshacki 2013 [8]	Yes	Yes	Yes	Yes	Yes	Yes	Yes	Yes	8	0	0	Good
Thorogood 2011 [9]	Yes	Yes	Yes	Yes	No	Yes	No	No	5	3	0	Poor
Turk 2017 [10]	Yes	Yes	Yes	Yes	Yes	Yes	Yes	Yes	8	0	0	Good
Vissers 2013 [11]	Yes	Yes	Yes	Yes	Yes	Yes	Yes	Yes	8	0	0	Good
Wewege 2017 [12]	Yes	Yes	Yes	Yes	No	Yes	Yes	Yes	7	1	0	Fair

Criteria: (1) Adequate research question; (2) Predefined and specified eligibility criteria; (3) Systematic search strategy; (4) Dual screening; (5) Dual quality assessment; (6) Listing of study characteristics and results; (7) Publication bias assessment; (8) Heterogeneity assessment.

References

1. Andreato LV, Esteves JV, Coimbra DR, Moraes AJP, de Carvalho T. The influence of high-intensity interval training on anthropometric variables of adults with overweight or obesity: a systematic review and network meta-analysis. *Obes Rev*. 2019;20(1):142-155. doi:10.1111/obr.12766

2. Batacan RB, Duncan MJ, Dalbo VJ, Tucker PS, Fenning AS. Effects of high-intensity interval training on cardiometabolic health: a systematic review and meta-analysis of intervention studies. *Br J Sports Med*. 2017;51(6):494-503. doi:10.1136/bjsports-2015-095841

3. Cheng CC, Hsu CY, Liu JF. Effects of dietary and exercise intervention on weight loss and body composition in obese postmenopausal women: a systematic review and meta-analysis. *Menopause*. 2018;25(7):772-782. doi:10.1097/GME.0000000000001085

4. Ismail I, Keating SE, Baker MK, Johnson NA. A systematic review and meta-analysis of the effect of aerobic vs. resistance exercise training on visceral fat. *Obes Rev*. 2012;13(1):68-91. doi:10.1111/j.1467-789X.2011.00931.x

5. Johansson K, Neovius M, Hemmingsson E. Effects of anti-obesity drugs, diet, and exercise on weight-loss maintenance after a very-low-calorie diet or low-calorie diet: a systematic review and meta-analysis of randomized controlled trials. *Am J Clin Nutr*. 2014;99(1):14-23. doi:10.3945/ajcn.113.070052

6. Mabire L, Mani R, Liu L, Mulligan H, Baxter D. The Influence of Age, Sex and Body Mass Index on the Effectiveness of Brisk Walking for Obesity Management in Adults: A Systematic Review and Meta-Analysis. *J Phys Act Health*. 2017;14(5):389-407. doi:10.1123/jpah.2016-0064

7. Sardeli AV, Komatsu TR, Mori MA, Gaspari AF, Chacon-Mikahil MPT. Resistance Training Prevents Muscle Loss Induced by Caloric Restriction in Obese Elderly Individuals: A Systematic Review and Meta-Analysis. *Nutrients*. 2018;10(4). doi:10.3390/nu10040423

8. Schwingshackl L, Dias S, Strasser B, Hoffmann G. Impact of different training modalities on anthropometric and metabolic characteristics in overweight/obese subjects: a systematic review and network meta-analysis. *PLoS One*. 2013;8(12):e82853. doi:10.1371/journal.pone.0082853

9. Thorogood A, Mottillo S, Shimony A, et al. Isolated aerobic exercise and weight loss: a systematic review and meta-analysis of randomized controlled trials. *Am J Med*. 2011;124(8):747-755. doi:10.1016/j.amjmed.2011.02.037

10. Turk Y, Theel W, Kasteleyn MJ, et al. High intensity training in obesity: a Meta-analysis. *Obes Sci Pract*. 2017;3(3):258-271. doi:10.1002/osp4.109

11. Vissers D, Hens W, Taeymans J, Baeyens J-P, Poortmans J, Van Gaal L. The effect of exercise on visceral adipose tissue in overweight adults: a systematic review and meta-analysis. *PloS One*. 2013;8(2):e56415. doi:10.1371/journal.pone.0056415

12. Wewege M, van den Berg R, Ward RE, Keech A. The effects of high-intensity interval training vs. moderate-intensity continuous training on body composition in overweight and obese adults: a systematic review and meta-analysis. *Obes Rev*. 2017;18(6):635-646. doi:10.1111/obr.12532
